# P-528. Opportunities for PrEP Awareness and Engagement: A Survey among Healthcare-Seeking Cisgender Women in the US

**DOI:** 10.1093/ofid/ofae631.727

**Published:** 2025-01-29

**Authors:** Tonia Poteat, Supriya Sarkar, Leigh Ragone, Kyli Gallington, Sonya Stanczyk, Patrick Daniele, Karin S Coyne, Vani Vannappagari

**Affiliations:** Duke University, Durham, North Carolina; ViiV Healthcare, Baltimore, Maryland; ViiV Healthcare, Baltimore, Maryland; Evidera, Sacramento, California; Evidera, Sacramento, California; Evidera, Sacramento, California; Evidera, Sacramento, California; ViiV Healthcare, Baltimore, Maryland

## Abstract

**Background:**

Cisgender women comprise 19% of new HIV diagnoses and account for 8% of HIV pre-exposure prophylaxis (PrEP) users in the US. These data highlight the need to close this gap, especially as new PrEP options, such as long-acting (LA) PrEP, are available. This study assessed PrEP awareness and interest among healthcare-seeking women.

**Methods:**

Cisgender women participated in an in-person survey at four health clinics in Kentucky, Seattle, St. Louis, and Philadelphia between Oct–Dec 2022. Inclusion criteria included: age ≥18 years; penetrative sex in the past six months; unknown or negative HIV status. The survey covered demographics, sexual and reproductive health, healthcare engagement, and HIV prevention, including PrEP awareness and interest. Descriptive statistics were calculated using SAS v9.4.

**Results:**

Among the 185 women completing the survey (median age: 28, women of color: 48%, single/dating: 69%), half (51%, n=95) had heard of daily oral (DO) PrEP, and 18% (n=33) had heard of LA PrEP prior to taking the survey; none were currently taking any form of PrEP. Regarding sexual practices, participants reported: no condom use during last sex (68%, n=126); ≥2 sexual partners in the last year (58%, n=108); ever received an HIV test (64%, n=119); STI diagnosis in the past two years (17%, n=32). Participants who saw a healthcare provider (HCP) in the past two years (n=174) reported discussing the following with their HCP: birth control (85%), sexual health (62%), STI testing (67%), and HIV testing (32%); only 5% of participants (n=9/185) reported ever discussing PrEP with an HCP. When asked about starting PrEP, 25% expressed interest in DO PrEP and 18% in LA PrEP. Among participants not interested in LA PrEP (n=104, 56%), the main reasons cited were: “*I don’t need it*” (60%) and “*I don’t think that I am at risk for HIV*” (55%). However, among women expressing interest in multiple PrEP options (n=42, 23%), 45% would choose to start LA PrEP over the other PrEP options provided.

**Conclusion:**

PrEP awareness and usage remain considerably low in the US among cisgender women who may benefit from PrEP. Efforts to increase awareness of PrEP among women in the US are needed, and newer PrEP options may provide HCPs opportunities to discuss PrEP in conjunction with other sexual and reproductive health topics.

**Disclosures:**

**Tonia Poteat, PhD, MPH, PA-C**, ViiV Healthcare: Grant/Research Support **Supriya Sarkar, PhD, MPH**, GSK: Stocks/Bonds (Public Company)|ViiV Healthcare: Full time employee **Leigh Ragone, MS**, GlaxoSmithKline: Stocks/Bonds (Public Company)|ViiV Healthcare: Full time employee **Kyli Gallington, MPH**, ViiV Healthcare: Advisor/Consultant **Patrick Daniele, MS**, GSK: Advisor/Consultant|ViiV Healthcare: Advisor/Consultant **Karin S. Coyne, PhD, MPH**, ViiV: Advisor/Consultant|ViiV: Grant/Research Support **Vani Vannappagari, MBBS, MPH, PhD**, GSK: Stocks/Bonds (Public Company)|ViiV Healthcare: Full time Employee|ViiV Healthcare: Stocks/Bonds (Public Company)

